# Effect of dolutegravir on folate, vitamin B12 and mean corpuscular volume levels among children and adolescents with HIV: a sub‐study of the ODYSSEY randomized controlled trial

**DOI:** 10.1002/jia2.26174

**Published:** 2023-09-27

**Authors:** Linda Namutebi Barlow‐Mosha, Grace Miriam Ahimbisibwe, Elizabeth Chappell, Pauline Mary Amuge, Annet Nanduudu, Elizabeth Kaudha, Timothy Amukele, David Balamusani, Bosco Kafufu, Audrey Nimwesiga, Hajira Kataike, Rosemary Namwanje, Gladys Kasangaki, Alice Mulindwa, Gerald Agaba Muzorah, Dickson Bbuye, Victor Musiime, Emmanuel Mujyambere, Mark Ssenyonga, Disan Mulima, Raymonds Crespo Kyambadde, Josephine Namusanje, Richard Isabirye, Mariam Nabalamba, Barbara Musoke Nakirya, Cissy Kityo, Adeodata R. Kekitiinwa, Carlo Giaquinto, Andrew Copp, Diana M. Gibb, Deborah Ford, Philippa Musoke, Anna Turkova, the ODYSSEY trial team

**Affiliations:** ^1^ Makerere University‐Johns Hopkins University (MU‐JHU) Research Collaboration Kampala Uganda; ^2^ MRC Clinical Trials Unit at UCL, Institute of Clinical Trials and Methodology London UK; ^3^ Baylor College of Medicine Children's Foundation‐Uganda Kampala Uganda; ^4^ Joint Clinical Research Centre Kampala Uganda; ^5^ Department of Pathology ICON Central Laboratories Inc Farmingdale New York USA; ^6^ Infectious Diseases Institute Core Laboratory Kampala Uganda; ^7^ Department of Paediatrics and Child Health Makerere University College of Health Sciences Kampala Uganda; ^8^ Department of Women and Child Health University of Padova Padova Italy; ^9^ UCL Great Ormond Street Institute of Child Health London UK

**Keywords:** adolescents, children, dolutegravir, folate, HIV, vitamin B12

## Abstract

**Introduction:**

Dolutegravir‐based antiretroviral therapy (ART) is the preferred antiretroviral treatment for children and adolescents living with HIV. A large surveillance study in Botswana previously raised concerns about an association between pre‐conception dolutegravir and neural tube defects. Before these concerns were subsequently resolved, we set up a sub‐study to look at the effect of dolutegravir on levels of folate and vitamin B12 in children and adolescents within the randomized ODYSSEY trial, as folate and vitamin B12 are known to play a crucial role in neural tube development.

**Methods:**

We conducted the sub‐study among Ugandan ODYSSEY participants and compared folate and vitamin B12 between children randomized to dolutegravir‐based ART (DTG) and non‐dolutegravir‐based standard‐of‐care treatment (SOC). Plasma folate was measured at enrolment and week 4 on stored samples; in addition, plasma and red blood cell (RBC) folate and vitamin B12 were assayed at week ≥96 in prospectively collected samples. RBC mean corpuscular volume (MCV) was measured 24‐weekly in all ODYSSEY participants. Samples analysed in the sub‐study were collected between September 2016 and October 2020.

**Results:**

A total of 229 children aged ≥6 years were included in the sub‐study with median age at trial enrolment of 12.3 (interquartile range [IQR] 9.0, 14.7) years, and CD4 count of 501 (IQR 228, 695); 112 (49%) children were male. Most participants (225/229, 98%) had plasma folate results at enrolment and 214 (93%) children had results available for RBC folate, vitamin B12 and plasma folate at week ≥96. MCV results were analysed on 679 children aged ≥6 years enrolled in ODYSSEY. At week 4, mean plasma folate was significantly higher in the dolutegravir arm than in SOC (difference [DTG‐SOC] 1.6 ng/ml, 95% CI 0.8, 2.3; *p*<0.001), and this difference persisted to week ≥96 (2.7 ng/ml, 95% CI 1.7, 3.7; *p*<0.001). Mean RBC folate at ≥96 weeks was also higher in the DTG arm (difference 73 ng/ml, 95% CI 3, 143; *p* = 0.041). There was no difference in the treatment arms for vitamin B12 levels at ≥96 weeks or change in MCV through trial follow‐up.

**Conclusions:**

Plasma and RBC folate levels were higher in children and adolescents receiving dolutegravir‐based ART than on other ART regimens. Further studies are needed to clarify the mechanisms of these interactions and the clinical implications of increased blood folate levels.

## 1 INTRODUCTION

Dolutegravir‐based antiretroviral therapy (ART) has become the preferred treatment option for children living with HIV due to superior efficacy and similar safety compared to non‐dolutegravir‐based ART [1].

A number of studies explored the teratogenic effects of dolutegravir. Pre‐clinical animal studies conducted as part of the dolutegravir development programme showed no effect on embryonic or foetal development [2]; however, recent animal and cell‐model studies suggested possible increased risks for embryonic and foetal toxicity [3, 4].

One of the mechanisms by which drugs may contribute to neural tube defects (NTDs) and other teratogenic defects is through interference with folate transport or its metabolism [5, 6, 7]. Vitamin B12 is an essential component of folate one‐carbon metabolism, responsible for the synthesis of the purine and pyrimidine components of DNA, and for methyl group donation to a range of methyltransferases. These events are critical for early embryonic and foetal development [8, 9]. DNA synthesis impairment due to folate and/or vitamin B12 deficiency also leads to the arrest of red blood cell (RBC) division, continuation of cellular enlargement and increased mean cell volume (MCV) [10]; thus, high MCV could be an indicator of folate or B12 deficiency. There are no data on the effect of dolutegravir on folate, B12 and MCV changes in children and adolescents receiving dolutegravir‐based ART.

The largest surveillance study on the use of dolutegravir in pregnancy was conducted in Botswana. The first report from the Tsepamo study in May 2018 suggested an increased risk of NTD among infants of women who conceived on dolutegravir compared to non‐dolutegravir regimens (4/426 [0.9%] vs. 14/11,300 [0.12%]; prevalence difference 0.82%, 95% confidence interval (CI) 0.24, 2.3) [11]. Reassuringly, the latest reported data from August 2014 through to March 2022 indicated no increased NTD prevalence in women conceiving on dolutegravir compared to non‐dolutegravir regimens (10/5860 deliveries [0.11%] vs. 25/23,664 [0.11%]; prevalence difference 0.00%, 95% CI −0.07, 0.10), and no increased risk of NTD compared to the general population (prevalence difference 0.04%; 95% CI −0.01, 0.13) [12].

Before the concerns regarding the possible association of pre‐conception dolutegravir with NTDs had been resolved, and considering that a substantial proportion of ODYSSEY participants would be older than 17 years by the end of the randomized phase, with pregnancies already reported in the trial [1], we considered the question on the dolutegravir effect on folate and B12 status was important in this population. We set up a sub‐study within the randomized ODYSSEY trial to compare the effect of dolutegravir‐based ART on levels of folate and vitamin B12 versus non‐dolutegravir‐based standard‐of‐care in children and adolescents in Uganda. As folate and vitamin B12 levels affect MCV, we compared MCV values between the dolutegravir and standard‐of‐care treatment arms for all the participants in the ODYSSEY study.

## 2 METHODS

ODYSSEY (NCT02259127) is an open‐label, 96‐week randomized, multicentre, non‐inferiority trial comparing dolutegravir‐based ART (DTG arm) with standard‐of‐care (SOC arm) in children starting first‐line ART (ODYSSEY A) or second‐line ART (ODYSSEY B) in Africa, Europe and Southeast Asia [1]. Children in the SOC arm received primarily efavirenz‐based first‐line ART or protease‐inhibitor‐based second‐line ART. The choice of nucleoside reverse transcriptase inhibitors (NRTI) backbones among abacavir, tenofovir or zidovudine, coupled with lamivudine or emtricitabine, was made according to World Health Organization or national guidelines. Patients were followed‐up until the last patient reached 96 weeks.

Children in three Ugandan sites (Joint Clinical Research Center, Baylor Uganda and Makerere University‐Johns Hopkins University Research Collaboration), aged ≥6 years, were enrolled in folate and B12 sub‐study and had folate and vitamin B12 levels measured and analysed by the randomized arm. MCV comparison was a secondary analysis for the sub‐study which allowed us to explore the differences between the randomized arms in the whole trial.

We limited the sub‐study to children ≥6 years, as the majority of children in the main ODYSSEY trial were 6 years and older (679/707 [96%]), and folate and B12 normal levels tend to be higher and have wider normal ranges in younger children [13]. The sub‐study was conceived after the Tsepamo study had reported their interim results and a safety alert regarding a possible association between dolutegravir and NTD risk was issued [11]. At the start of the sub‐study, most ODYSSEY participants were in their third year of follow‐up, and RBC folate and vitamin B12 samples were collected prospectively at their next visit ≥96 weeks after randomization in the three Ugandan sites (as no suitable samples were stored). Plasma folate was measured on plasma samples stored at trial enrolment, week 4 and week ≥96 (week ≥96 was the same timepoint as for RBC folate and vitamin B12 for each participant). We measured plasma and RBC folate as indicators of short‐term and long‐term folate stores, respectively [9]. MCV values were measured at enrolment and weeks 4, 24, 48, 72 and 96. Samples analysed for MCV, folate or B12 were collected between 20 September 2016 and 19 October 2020. Plasma and RBC folate and serum B12 were analysed in the Infectious Disease Institute Core laboratory, Kampala, using validated Elecys assays [14, 15, 16]. MCV was assessed as part of routine full blood count blood tests done in local ODYSSEY site laboratories.

For the sub‐study sample size calculation, we considered a difference of half a standard deviation or more between randomized groups in RBC folate at week ≥96 and/or plasma folate at week 4 (adjusting for baseline) as clinically relevant. We estimated we would require 140 children to show these differences with 80% power, at a 5% level of significance, assuming a correlation between pre‐ and post‐treatment levels of plasma folate of 0.3, and allowing for 10% unanalysable samples. The sample size calculation is described in the ODYSSEY protocol [1].

In statistical analysis, laboratory measurements below the 1st percentile were recoded as equal to the 1st percentile, and above the 99th percentile as equal to the 99th percentile. Linear regression was used to compare cross‐sectional RBC folate and vitamin B12 between arms at week ≥96, and change from enrolment (adjusted for baseline) in plasma folate to weeks 4 and ≥96 and MCV to weeks 4, 24, 48, 72 and 96. Estimates were adjusted for site, sample date (using restricted cubic splines with knots at the 10th, 50th and 90th percentiles, included because of an observed non‐linear trend over time by sample date), time between sample taken and sample analysis, and randomization stratification factors (first‐/second‐line ART, site availability of routine resistance testing and NRTI backbone chosen pre‐randomization). Heterogeneity of treatment effects by first‐ and second‐line and by site were assessed using interaction terms.

Zidovudine competes with natural deoxynucleoside triphosphates causing impairment of mitochondrial DNA biosynthesis and mitochondrial dysfunction, which leads to arrested cell growth of erythrocyte precursors and macrocytosis [17]. Due to this known association between MCV levels and zidovudine use, treatment effects on MCV were evaluated in the following (non‐overlapping) groups: (i) children starting second‐line ART who had zidovudine in the 14 days prior to randomization and whose initial trial regimen did not contain zidovudine; (ii) children starting second‐line ART who did not have zidovudine in the 14 days prior to randomization and whose initial trial regimen contained zidovudine; (iii) children starting first‐line ART whose initial regimen did not contain zidovudine; and (iv) children starting second‐line ART whose initial second‐line regimen did not contain zidovudine and who had no zidovudine in the 14 days prior to treatment switch. Of note, we had only three children starting ZDV‐containing first‐line ART, and they were excluded from this analysis.

In sensitivity analyses of plasma and RBC folate and of serum vitamin B12, data in children with a treatment change prior to outcome measure were excluded, with treatment change defined as: (i) any time off third agent in the 3 months prior to the sample collection (where a third agent is a third antiretroviral drug co‐administered with two NRTIs used in the ART regimen), or (ii) time on non‐dolutegravir regimen for those in the DTG arm or on dolutegravir for those in the SOC arm prior to the sample collection. An interaction term between body weight at week ≥96 as a continuous covariate and treatment effect was considered as an indicator of the difference by dolutegravir dose per kilogram (99% of children were receiving the adult 50 mg dose).

The proportions of children with levels of plasma folate and RBC folate below and above the expected ranges were compared between trial arms, based on the lower and upper end of the reference ranges (normal ranges 3.89 to 26.8 ng/ml for plasma folate and 523 to 1257 ng/ml for RBC folate) [14, 15]. Logistic regression models (one comparing above vs. below the upper reference limit, and one above vs. below the lower reference limit) were used, adjusted for first‐ and second‐line ART.

Caretakers and children gave written informed consents and assents as appropriate for participation in the ODYSSEY trial and the sub‐study. The folate and B12 sub‐study was included in the ODYSSEY main protocol v5.0, which was approved by ethics committees for all ODYSSEY trial sites (Germany, Thailand, Portugal, Spain, South Africa, Uganda, UK and Zimbabwe).

## 3 RESULTS

A total of 229 children aged ≥6 years were included for folate and vitamin B12 measurements (Table 1). Seventy‐five (33%) initiated first‐line treatment (ODYSSEY A), and all 41 randomized to SOC started efavirenz‐based ART. One hundred fifty‐four (67%) initiated second‐line treatment (ODYSSEY B); of 74 children randomized to SOC, 46 (62%) started ART based on ritonavir‐boosted lopinavir, 27 (36%) atazanavir and 1 (1%) efavirenz. The median age and CD4 count at trial enrolment were 12.3 (IQR, interquartile range, 9.0, 14.7; range 6.0, 18.0) years and 501 (IQR 228, 795) cells/mm^3^, respectively, with 112 (49%) male children.

**Table 1:** Participant characteristics at enrolment in the ODYSSEY trial

	Enrolled in folate sub‐study				
	JCRC (*N* = 72)	Baylor (*N* = 110)	MUJHU (*N* = 47)	Total (*N* = 229)	All children in ODYSSEY enrolled ≥6 years^a^ (*N* = 679)
*n* (%) or median (IQR)					
Arm					
DTG	36 (50%)	52 (47%)	26 (55%)	114 (50%)	335 (49%)
SOC	36 (50%)	58 (53%)	21 (45%)	115 (50%)	344 (51%)
ODYSSEY A (first‐line)	12 (17%)	62 (56%)	1 (2%)	75 (33%)	298 (44%)
NRTI backbone					
ABC & 3TC	5 (42%)	46 (74%)	1 (100%)	52 (69%)	241 (81%)
TDF & 3TC/FTC	7 (58%)	16 (26%)	0	23 (31%)	55 (18%)
ZDV & 3TC	0	0	0	0	2 (1%)
Other	0	0	0	0	1 (1%)
Third agent among those on SOC	(*n* = 6)	(*n* = 35)	(*n* = 0)	(*n* = 41)	(*n* = 151)
EFV	6 (100%)	35 (100%)	−	41 (100%)	139 (92%)
Other	0	0	−	0	12 (8%)
ODYSSEY B (second‐line)	60 (83%)	48 (44%)	46 (98%)	154 (67%)	381 (56%)
NRTI backbone					
ABC & 3TC	19 (32%)	20 (42%)	21 (46%)	60 (39%)	202 (53%)
TDF & 3TC/FTC	22 (27%)	25 (52%)	20 (43%)	67 (43%)	106 (28%)
ZDV & 3TC	19 (32%)	3 (6%)	5 (11%)	27 (18%)	70 (18%)
Other	0	0	0	0	3 (1%)
Third agent among those on SOC	(*n* = 30)	(*n* = 23)	(*n* = 21)	(*n* = 74)	(*n* = 193)
ATV	15 (50%)	12 (52%)	0	27 (36%)	48 (25%)
EFV	0	1 (4%)	0	1 (1%)	5 (3%)
LPV/r	15 (50%)	10 (43%)	21 (100%)	46 (62%)	138 (72%)
DRV	0	0	0	0	2 (1%)
Sex					
Male	40 (56%)	47 (43%)	25 (53%)	112 (49%)	348 (51%)
Female	32 (44%)	63 (57%)	22 (47%)	117 (51%)	331 (49%)
Age (years)	13.3 (10.8, 15.9)	11.7 (7.9, 14.4)	11.8 (9.1, 14.0)	12.3 (9.0, 14.7)	12.3 (9.4, 15.0)
CD4 count (cells/mm^3^)	442 (170, 682)	561 (335, 813)	492 (144, 893)	501 (228, 795)	444 (221, 673)
BMI‐for‐age z‐score	−0.9 (−1.5, −0.5)	−0.4 (−1.0, 0.4)	−0.5 (−1.2, 0.0)	−0.6 (−1.3, 0.1)	−0.6 (−1.4, 0.0)
Haemoglobin (g/dl)	12.1 (11.4, 13.1)	12.0 (11.0, 12.8)	12.5 (11.7, 13.3)	12.1 (11.2, 13.0)	11.9 (10.9, 13.0)

Abbreviations: ABC, abacavir; ATV, atazanavir; Baylor, Baylor College of Medicine Children's Foundation; BMI, body mass index; DRV, darunavir; DTG, dolutegravir; EFV, efavirenz; FTC, emtricitabine; IQR, interquartile range; JCRC, Joint Clinical Research Centre; LPV/r, lopinavir/ritonavir; MUJHU, Makerere University‐Johns Hopkins University (MU‐JHU) Research Collaboration; NRTIs, nucleotide reverse transcriptase inhibitors; SOC, standard‐of‐care; TDF, tenofovir disoproxil fumarate; ZDV, zidovudine; 3TC, lamivudine.

^a^ Excludes two children enrolled in ODYSSEY ≥6 years who had no MCV data.

Of the 229 children included, 225 (98%) had an available plasma folate result at enrolment. By week 4, 226 (99%) children were still in follow‐up, of whom 218 (96%) had an available plasma folate result; three met the definition for treatment change prior to week 4 (1 DTG, 2 SOC). Two hundred and fifteen (94%) children had samples collected for prospective evaluation of folate and vitamin B12 at median 144 weeks (IQR 132, 167) post randomization, and of these, 214 children had available results for RBC folate, vitamin B12 and plasma folate (“week ≥96 measurement”). Of these 214 children, 9 (7 DTG, 2 SOC) met the definition of a treatment change prior to their ≥96 week sample being taken.

Overall, 679 children aged ≥6 years were enrolled in the ODYSSEY trial and had available MCV data (at enrolment, and at least one measure subsequently); characteristics were generally similar to those enrolled in the sub‐study in the three Ugandan sites, although more children in the Ugandan sites were on second‐line ART.

### 3.1 Plasma folate

**Figure 1:**
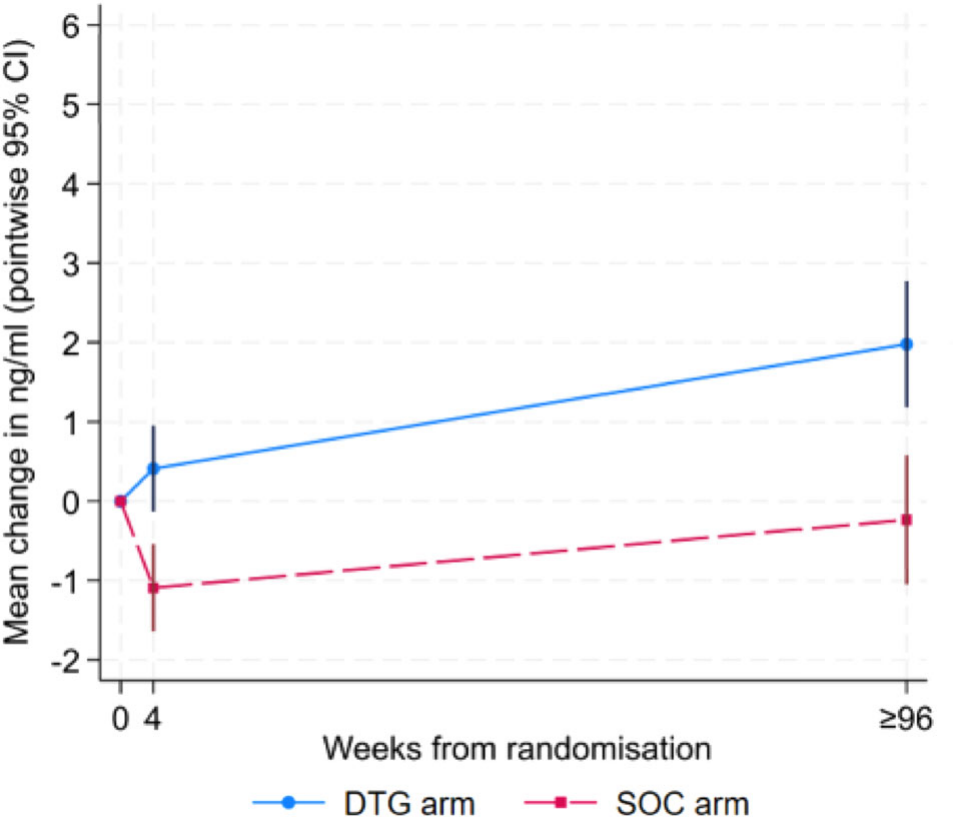
Change in plasma folate from baseline to week 4 and week ≥96. CI, confidence interval; DTG, dolutegravir; SOC, standard‐of‐care.

In adjusted analysis, at 4 weeks, mean plasma folate was higher in the DTG arm than in SOC (difference [DTG‐SOC] 1.6 ng/ml, 95% CI 0.8, 2.3; *p*<0.001), and this difference increased to week ≥96 (difference 2.7 ng/ml, 95% CI 1.7, 3.7; *p*<0.001) (Table 2 and Figure 1). Results were similar excluding those with treatment changes (week 4 difference 1.4 ng/ml, 95% CI 0.6, 2.2; *p*<0.001; week 96 difference 2.3 ng/ml, 95% CI 1.2, 3.4; *p*<0.001). There was no evidence of heterogeneity in treatment effect by weight at week ≥96, explored by looking at the difference by dolutegravir dose per kilogram (change in plasma folate with weight per kg in the DTG arm: −0.04 ng/ml, 95% CI −0.10, 0.03; in the SOC arm: −0.03 ng/ml, 95% CI −0.10, 0.04; interaction *p* = 0.82). A higher proportion of those on SOC had plasma folate levels that dropped below the normal range (17/109 [16%] DTG and 38/105 [36%] SOC, odds ratio 0.30, 95% CI 0.15–0.59, *p*<0.001), but there was no difference in the proportion above the normal range (0/109 [0%] DTG and 1/105 [1%] SOC) (Table 2).

**Table 2:** Difference in change in plasma folate and red blood cell mean cell volume from baseline, and cross‐sectional red blood cell folate and serum vitamin B12 at ≥96 weeks, in the DTG arm compared to the SOC arm

Measure^a^	DTG arm			SOC arm			Difference adjusted for first‐/second‐line and baseline (where relevant) (DTG‐SOC)^b^			Fully adjusted difference (DTG‐SOC)^c^			Arm × site interaction	Arm × A/B interaction
	*n*	mean	SE	*n*	mean	SE	mean	95% CI	*p*	mean	95% CI	*p*	*p*	*p*
Plasma folate, ng/ml:														
Baseline	112	5.8	0.3	113	6.3	0.3	−	−	−	−	−	−	−	−
Week 4	111	6.3	0.3	107	5.1	0.3	−	−	−	−	−	−	−	−
Change to week 4	110	0.4	0.3	107	−1.1	0.3	1.5	0.7, 2.3	<0.001	1.6	0.8, 2.3	<0.001	0.65	0.41
Week ≥96	109	8.0	0.4	105	5.8	0.4	−	−	−	−	−	−	−	−
Change to week ≥96	108	2.0	0.4	103	−0.2	0.4	2.3	1.2, 3.4	<0.001	2.7	1.7, 3.7	<0.001	0.86	0.60
RBC folate at week ≥96, ng/ml	109	888	29	105	855	28	36	−44, 115	0.38	73	3, 143	0.041	0.82	0.95
Vitamin B12 at week ≥96, pg/ml	109	478	21	105	504	26	−25	−90, 41	0.46	−26	−91, 39	0.42	0.57	0.50
MCV, fl:														
Baseline	335	86.9	0.6	344	86.4	0.6	−	−	−	−	−	−	−	−
Week 4	331	86.6	0.6	338	86.0	0.6	−	−	−	−	−	−	−	−
Change to week 4	331	−0.2	0.2	338	−0.4	0.2	0.2	−0.4, 0.7	0.53	0.2	−0.4, 0.7	0.56	−	0.19
Week 24	331	85.5	0.5	336	86.3	0.5	−	−	−	−	−	−	−	−
Change to week 24	331	−1.3	0.4	336	−0.3	0.4	−1.0	−2.1, 0.1	0.09	−1.0	−2.1, 0.0	0.06	−	0.26
Week 48	330	86.3	0.5	327	86.6	0.5	−	−	−	−	−	−	−	−
Change to week 48	330	−0.5	0.4	327	0.0	0.4	−0.5	−1.7, 0.7	0.40	−0.5	−1.6, 0.7	0.40	−	0.45
Week 72	329	86.8	0.5	325	87.0	0.5	−	−	−	−	−	−	−	−
Change to week 72	329	0.1	0.5	325	0.3	0.5	−0.3	−1.5, 1.0	0.65	−0.3	−1.5, 0.9	0.62	−	0.76
Week 96	325	87.1	0.5	322	87.7	0.5	−	−	−	−	−	−	−	−
Change to week 96	325	0.4	0.5	322	1.0	0.5	−0.7	−1.9, 0.6	0.30	−0.7	−1.9, 0.6	0.29	−	0.66

Abbreviations: CI, confidence interval; DTG, dolutegravir; MCV, mean cell volume; RBC, red blood cell; SE, standard error; SOC, standard‐of‐care.

^a^ Normal ranges: plasma folate 3.89–26.8 ng/ml [15], RBC folate 523–1257 ng/ml [14], B12 179–771 pg/ml [16] and MCV 80–95 fl [18].

^b^ Normal regression adjusted for first‐ versus second‐line. Change in plasma folate and MCV were also adjusted for baseline value.

^c^ Normal regression also adjusted for first‐ versus second‐line, availability of routine resistance testing, NRTI backbone, and site, date of sample and time from sample taken to analysis (plasma folate, RBC folate and vitamin B12 only), and baseline value (for plasma folate and MCV).

### 3.2 RBC folate

In adjusted analysis, mean RBC folate after more than 96 weeks in the trial was higher in the DTG arm than in SOC (difference 73 ng/ml; 95% CI 3, 143; *p* = 0.041) (Table 2). RBC folate levels varied by site, although there was no evidence of heterogeneity of treatment effect (*p* = 0.82). When excluding children with treatment changes, the difference between trial arms was similar but not significant (difference 63 ng/ml, 95% CI −7, 133; *p* = 0.08). There was no evidence of a difference in treatment effect by weight (change in RBC folate with weight per kg in the DTG arm: −3.49 ng/ml, 95% CI −7.67, 0.70; in the SOC arm: −3.74 ng/ml, 95% CI −8.22, 0.73; interaction *p* = 0.93). There was no difference in proportions of children with RBC folate levels below or above the normal range (8/109 [7%] DTG and 8/105 [8%] SOC, odds ratio 0.94, 95% CI 0.34, 2.62, *p* = 0.91; 16/109 [15%] DTG and 10/105 [10%] SOC, odds ratio 1.66, 95% CI 0.72, 3.86, *p* = 0.24 respectively).

### 3.3 Vitamin B12

There was no difference in vitamin B12 levels between the randomized arms at ≥96 weeks in the trial (difference −26 pg/ml, 95% CI −91, 39; *p* = 0.42). Levels varied by site, although there was no evidence of heterogeneity of treatment effect (*p* = 0.57). Results were similar after the exclusion of children with treatment changes (difference −36 pg/ml, 95% CI −103, 30; *p* = 0.29). There was no evidence of a difference in treatment effect by weight (change in B12 with weight per kg in the DTG arm: 1.72 pg/ml, 95% CI −2.33, 5.79; in the SOC arm: −2.08 pg/ml, 95% CI −6.43, 2.27; interaction *p* = 0.15).

### 3.4 Red blood cell MCV

**Figure 2:**
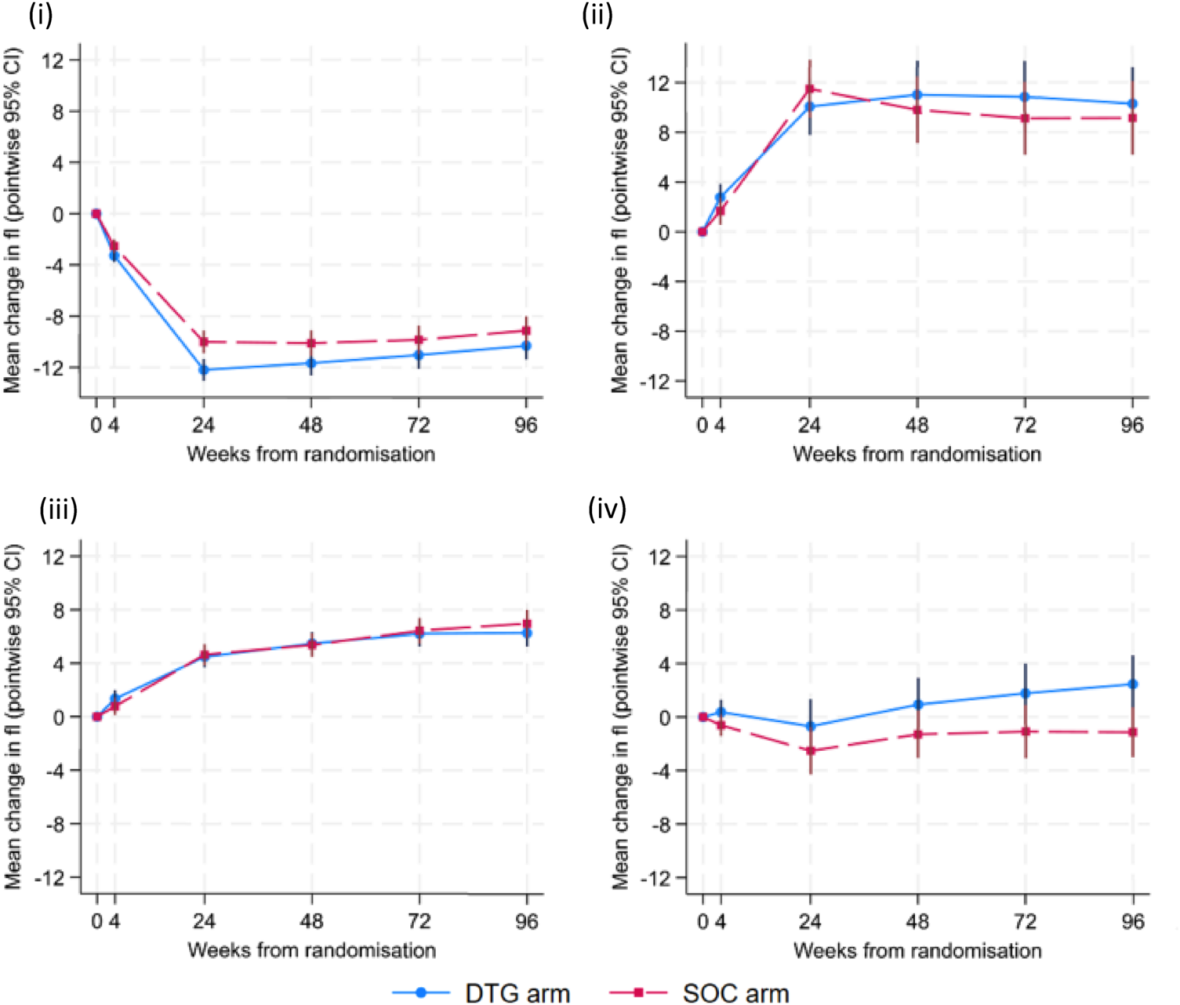
Change in red blood cell mean cell volume to week 96, by treatment arm and ZDV exposure. (i) Children starting second‐line ART who had zidovudine in the 14 days prior to randomization and whose initial trial regimen did not contain zidovudine; (ii) children starting second‐line ART who did not have zidovudine in the 14 days prior to randomization and whose initial trial regimen contained zidovudine; (iii) children starting first‐line whose initial first‐line regimen did not contain zidovudine; and (iv) children starting second‐line whose initial second‐line regimen did not contain zidovudine and who did not have zidovudine in the 14 days prior to treatment switch. Participants in ODYSSEY A whose initial regimen included zidovudine are not included due to small numbers (*n* = 3). ART, antiretroviral therapy; CI, confidence interval; DTG, dolutegravir; SOC, standard‐of‐care; ZDV, zidovudine.

There was no evidence of a difference in change in MCV from enrolment (adjusted for baseline) by treatment arm. However, as expected, different trends were observed by zidovudine use (Table 2 and Figure 2), with an initial decrease in MCV observed among those who were switched off zidovudine at randomization, and an initial increase observed among those who began zidovudine at randomization.

## 4 DISCUSSION

This is the first paediatric study to compare folate, vitamin B12 and MCV levels in children and adolescents receiving dolutegravir versus non‐dolutegravir ART. We found the levels of plasma folate at weeks 4 and ≥96 and RBC folate at week ≥96 were higher in those on dolutegravir than SOC, with fewer children in the DTG arm having plasma folate levels below the normal range at week ≥96. There was also a marginally higher proportion of children and adolescents on dolutegravir with RBC folate above the normal range at ≥96 weeks (15% DTG and 10% SOC). However, we found no evidence for a difference in treatment effect by weight for plasma or RBC folate, which would have suggested a dose−response effect for dolutegravir. We showed no difference between treatment arms in vitamin B12 at week ≥96 or in change in MCV from baseline over follow‐up.

These results are in line with those observed in the folate sub‐study of the randomized ADVANCE trial which compared serum folate in women receiving dolutegravir‐based regimens with those on an efavirenz‐based regimen [7]. This study showed the serum folate concentrations were higher in females receiving dolutegravir compared to efavirenz and a smaller proportion of women on dolutegravir experienced folate deficiency at 24 weeks (26/281 [9%] on dolutegravir vs. 38/125 [30%] on efavirenz; *p*<0.001) [7]. In the ADVANCE study, higher folate levels among women taking dolutegravir were postulated to be due to dolutegravir reducing the cellular uptake of folate by folate receptors. In support of this hypothesis, Cabrera et al. showed that dolutegravir is a partial antagonist of folate receptor FOLR1 and further suggested that folate supplementation may ameliorate this effect [5]. Although such a mechanism could account for the higher plasma folate concentrations, it would not account for the higher RBC folate concentrations observed in the present study. Mohan et al. [3] found no reduction in total foetal folate and even an increase in mouse foetuses exposed to supratherapeutic dolutegravir dose, which would go against the hypothesis of dolutegravir reducing folate cellular uptake. Alternative dolutegravir‐related mechanisms of increased plasma and cellular folate could be increased food uptake due to increased appetite or increased folate absorption from the gut.

Folate uptake from the intestine could be enhanced by dolutegravir through its effect on the proton‐coupled folate transporter (PCFT), which is the main folate transporter working at acid pH [19]. However, in placental cells, Gilmore et al. [6] found that PCFT transport function was diminished by dolutegravir. If this is also happening in the gut (so far unknown) that would lead to the reduced uptake, and therefore, cannot explain the increased folate levels in children.

In the ODYSSEY main trial, children in the dolutegravir arm gained 1 kg more weight on average and grew better [1], and it is possible that they had better appetite and ate more and, therefore, had higher folate intake. Children in ODYSSEY on dolutegravir had also slightly higher haemoglobin at 96 weeks compared to SOC (adjusted difference 0.29 g/dl, 95% CI 0.11, 0.47; *p* = 0.002) (analysed as part of the main trial; personal communication DF, 13 November 2022). This could be related to the higher plasma and RBC folate levels, as folate stimulates the production of red cells, and there is an established correlation between haemoglobin and folate levels [20].

The results of our sub‐study provide reassuring results that dolutegravir‐based ART is not associated with low folate or B12 levels. However, the study showed that dolutegravir leads to increased plasma and RBC folate levels, although the mechanisms are poorly understood. Overall, higher haemoglobin and a lower number of children with insufficient plasma folate levels in the DTG arm compared to SOC indicate a better haematological health in children and adolescents receiving DTG‐based treatment.

Dolutegravir has been associated with increased weight gain, obesity and new‐onset type 2 diabetes mellitus among adults, including pregnant women initiating or transitioning to dolutegravir‐based ART [21, 22]. Interestingly, some studies have suggested an association between maternal folate and vitamin B12 levels and gestational diabetes [23, 24, 25]. Given the observed elevation of folate in ODYSSEY and ADVANCE, the association between dolutegravir and increased weight gain and diabetes through modified folate metabolism should be further explored.

The sub‐study was constrained to three urban research centres in Uganda, hence this may limit the generalizability of the results. Another limitation is that RBC folate was not collected at baseline, and therefore, we cannot evaluate the difference in RBC folate change over time between DTG and SOC arms. However, participants were in a randomized controlled trial, and baseline RBC folate would likely be similar between arms, enabling a fair comparison between dolutegravir‐ and non‐dolutegravir‐based ART regimens. All assessments were performed in one laboratory using validated assays which minimizes measurement bias.

## 5 CONCLUSIONS

In conclusion, we found that plasma and RBC folate levels were higher in children receiving dolutegravir compared to other ART regimens. Further studies are needed to clarify the mechanism of this interaction and possible clinical implications.

## COMPETING INTERESTS

AT has received funding for serving on a ViiV Healthcare advisory board with payment made to the employer. Other authors have no conflict of interest to declare.

## AUTHORS’ CONTRIBUTIONS

LNB‐M, AT and DF conceived the study. EC and DF analysed the data. LNB‐M, GMA and EC wrote the first drafts of the manuscript. LNB‐M, GMA, EC, DF, PM and AT reviewed the results and revised the manuscript. All authors made crucial revisions to and approved the final manuscript.

## FUNDING

The study was sponsored by the Penta Foundation and funded by ViiV Healthcare and the UK Medical Research Council (grant MC_UU_00004/03).

## Supporting information



Supporting information

## Data Availability

The data that support the findings of this study are available on request from the corresponding author. The data are not publicly available due to privacy or ethical restrictions.
