# Supplementary material for: Effect of dolutegravir on folate, vitamin B12 and mean corpuscular volume levels among children and adolescents with HIV: a sub‐study of the ODYSSEY randomized controlled trial
Source: J Int AIDS Soc. 2023 Sep 27;26(9):e26174. doi: 10.1002/jia2.26174 (PMC10534059; doi:10.1002/jia2.26174)
Supplement: Supplementary file 1 — Supporting information [file JIA2-26-e26174-s001.docx]

The ODYSSEY Trial Team

**Penta Foundation:** Carlo Giaquinto, Tiziana Grossele, Daniel Gomez-Pena, Davide Bilardi, Giulio Vecchia

*Clinical Trial Units*

**MRC CTU at UCL:** Shabinah S. Ali, Abdel Babiker, Shazia Begum, Chiara Borg, Anne-Marie Borges Da Silva, Joanna Calvert, Man Chan, Nimisha Dudakia, Deborah Ford, Joshua Gasa, Diana M. Gibb, Lily Houlden, Khadija Ibrahim, Nasir Jamil, Sarah Lensen, Emma Little, Fatima Mohamed, Samuel Montero, Cecilia L. Moore, Rachel Oguntimehin, Anna Parker, Reena Patel, Tasmin Phillips, Tatiana Sarfati, Karen Scott, Clare Shakeshaft, Moira Spyer, Margaret Thomason, Anna Turkova, Rebecca Turner, Nadine Van Looy, Ellen White, Ian White, Kaja Widuch, Helen Wilkes, Ben Wynne

**INSERM SC-10-US19—ANRS:** Alexandra Compagnucci, Yacine Saidi, Yoann Riault, Alexandra Coelho, Laura Picault, Christelle Kouakam

**PHPT:** Tim R. Cressey, Suwalai Chalermpantmetagul, Dujrudee Chinwong, Gonzague Jourdain, Rukchanok Peongjakta, Praornsuda Sukrakanchana, Wasna Sirirungsi

*Trial sites*

**Joint Clinical Research Centre, Uganda:** Cissy M. Kityo, Victor Musiime, Elizabeth Kaudha, Annet Nanduudu, Emmanuel Mujyambere, Paul Ocitti Labeja, Charity Nankunda, Juliet Ategeka, Peter Erim, Collin Makanga, Esther Nambi, Abbas Lugemwa, Lorna Atwine, Edridah Keminyeto, Deogratiuos Tukwasibwe, Shafic Makumbi, Emily Ninsiima, Mercy Tukamushaba, Rogers Ankunda, Ian Natuhurira, Miriam Kasozi, Baker Rubinga, Diana Antonia Rutebarika, Rashidah Nazzinda, Shamim Nakabuye, Julius Tumusiime, Alice Mulindwa, Ritah Mbabazi, Milly Ndigendawani, Edward Bagirigomwa, Eddie Rubanga, David Eram, Maria Nannungi, Chrispus Katemba, Disan Mulima, Josephine Namusanje, Mariam Nabalamba, Priscilla Kyobutungi, Phyllis Mwesigwa Rubondo, Robinah Kibenge, Claire Nasaazi, Basiimwa Roy Clark, Enock Babu, Alex Musiime, Faith Mbasani, Martin Ojok, Odoch Denis, David Baliruno, Katabalwa Juliet, Benson Ouma, Barbara Ainebyoona, Mariam Naabalamba, Diana A. Rutebarika, Alex V. Musiime, Josephine Kobusungye, Ezra Lutalo

**Baylor College of Medicine Children’s Foundation, Uganda:** Adeodata R. Kekitiinwa, Pauline Amuge, Dickson Bbuye, Justine Nalubwama, Winnie Akobye, Muzamil Nsibuka Kisekka, Anthony Kirabira, Gloria Ninsiima, Sylvia Namanda, Gerald M. Agaba, Immaculate Nagawa, Annet Nalugo, Florence Namuli, Rose J. Kadhuba, Rachael K. Namuddu, Lameck Kiyimba, Angella Baita, Eunice Atim, Olivia Kobusingye, Clementine Namajja, Africanus Byaruhanga, Rogers Besigye, Herbert Murungi, Geoffrey Onen, Lawrence Lekku, Judith Tikabibamu, Henry Balwa

**MUJHU Research Collaboration, Uganda:** Philippa Musoke, Linda Barlow-Mosha, Grace Ahimbisibwe, Rosemary Namwanje, Hajira Kataike, Mark Ssenyonga, Brenda Kakayi, Rebecca Sakwa, Sarah Nakabuye, Barbara Musoke Nakirya, Gladys Kasangaki, Raymonds Kyambadde, David Balamusani, Winnie Nansamba, Stella Nalusiba,  Emmanuel Mayanja, Richard Isabirye, Erinah Kyomukama, Rebecca Wampamba, Mildred Kabasonga, Zaam Zinda Nakawungu, Sarah Babirye, Olivia Kaboggoza, Juliet Nanyonjo, Joanita Nankya Baddokwaya, Alice Elwana, Winfred Kaahwa, Bosco Kafufu, Emmanuel Hakiza, Maria Musisi, Paula Namayanja, Maria Gorreti Nakalema, Robert Serunjogi, Monica Etima, Phionah Kibalama, Joel Maena, Agnes Mary Mugagga, Annet Miwanda, Monica Nolan.

**UZCRC, Zimbabwe:** James Hakim, Hilda Mujuru, Kusum Nathoo, Mutsa Bwakura-Dangarembizi, Ennie Chidziva, Shepherd Mudzingwa, Secrecy Gondo, Godfrey Musoro, Vivian Mumbiro, Gloria Tinago, Shirley Mutsai, Joy Chimanzi, Columbus Moyo, Ruth Nhema, Misheck Nkalo Phiri, Stuart Chitongo, Joshua Choga, Joyline Bhiri, Wilber Ishemunyoro, Makhosonke Ndlovu, Moses Chitsamatanga, Pia Ngwaru, Tsitsi Gwenzi, Wendy Mapfumo, Dorothy Murungu, Trust Mukanganiki, Prosper Dube, Tapiwa Gwaze, Farai Matimba, Tawona Mudzviti, Zivai Mupambireyi, Sibusisiwe Weza, Cleopatra Langa, Sandra Musarurwa, Shamiso Gwande

**FAM-CRU, South Africa:** Mark F. Cotton, Anita Janse van Rensburg, Marlize Smuts, Catherine Andrea, Sumaya Dadan, Sonja Pieterse, Vinesh Jeaven, Candice Makola, George Fourie, Kurt Smith, Els Dobbels, Peter Zuidewind, Hesti Van Huyssteen, Mornay Isaacs, Georgina Nentsa, Thabisa Ncgaba, Candice MacDonald, Maria Bester, Wilma Orange, Ronelle Arendze,  Mark Mulder, Lucille Malgraaf, Ashley Harley, Yasmeen Akhalwaya

**PHRU, South Africa:** Avy Violari, Nastassja Ramsagar, Afaaf Liberty, Ruth Mathiba, Mandisa Nyati, Haseena Cassim, Lindiwe Maseko, Nkata Kekane, Busi Khumalo, Mirriam Khunene, Noshalaza Sbisi, Jackie Brown, Tryphina Madonsela, Nokuthula Mbadaliga, Zaakirah Essack, Reshma Lakha, Aasia Vadee, Derusha Frank, Nazim Akoojee, Maletsatsi Monametsi, Gladness Machache, Yolandie Fourie, Anusha Nanan-kanjee, Juan Erasmus, Angelous Mamiane, Tseleng Daniel, Fatima Mayat, Nomfundo Maduna, Patsy Baliram, Sibongile Sithebe, Emily Lebotsa, Siphiwe Mkhize

**Klerksdorp Tshepong Hospital Complex, South Africa:** Ebrahim Variava, Modiehi Rakgokong, Dihedile Scheppers, Tumelo Moloantoa, Abdul Hamid Kaka, Tshepiso Masienyane, Akshmi Ori, Kgosimang Mmolawa, Pattamukkil Abraham.

**Durban International Clinical Research Site, South Africa:** Moherndran Archary, Rosie Mngqibisa, Rejoice Mosia, Sajeeda Mawlana, Rashina Nundlal, Penelope Madlala, Allemah Naidoo, Sphiwee Cebekhulu, Petronelle Casey,  Subashinie Sidhoo, Minenhle Chikowore, Lungile Nyantsa, Sheleika Singh

**AHRI, South Africa:** Nigel Klein, Osee Behuhuma, Olivier Koole, Kristien Bird, Nomzamo Buthelezi, Mumsy Mthethwa, Gugu Gasa, Siva Danaviah and Theresa Smit

**PHPT CTU:** Tim R. Cressey, Suwalai Chalermpantmetagul, Gonzague Jourdain, Nicole Ngo Giang Huong, Dujrudee Chinwong, Chalermpong Saenjum, Rukchanok Peongjakta, Pra-ornsuda Sukrakanchana, Woottichai Khamduang, Laddawan Laomanit, Ampika Kaewbundit, Jiraporn Khamkon, Kanchana Than-in-at, Sanuphong Chailert, Worathip Sripaoraya, Nitinart.krueduangkam, Namthip Kruenual, Warunee Khamjakkaew, Soraya Klinprung, **Prapokklao Hospital:** Chaiwat Ngampiyaskul, Pisut Greetanukroh, Praechadaporn Khannak, Pathanee Tearsansern, Wanna Chamjamrat, **Phayao Hospital, Thailand:** Nuttawut Chanto, Thitiwat Thapwai, Khanungnit Thungkham, Patcharee Puangmalai, Chutima Ruklao, **Chiangrai Prachanukroh Hospital, Thailand:** Pradthana Ounchanum, Suwimon Khusuwan, Sukanda Denjanta, Yupawan Thaweesombat, Jutarat Thewsoongnoen, Kanyanee Kaewmamueng, Phakamas Kamboua, Supawadee Pongprapass (Sangjan), Warunee Srisuk, Areerat Kongponoi, Juthamas Limplertjareanwanich, **Nakornping Hospital, Thailand:** Suparat Kanjanavanit, Prattana Leenasirimakul, Chayakorn Saewtrakool, Pacharaporn Yingyong, Duangrat Chutima (Suwan), Raungwit Junkaew, Orapin Khatngam, Thannapat Chankun, **Khon Kaen Hospital, Thailand:** Ussanee Srirompotong, Patamawadee Sudsaard, Sookpanee Wimonklang, Turian Petpranee, **Mahasarakam Hospital, Thailand:** Sathaporn Na-Rajsima, Pattira Runarassamee, Nuananong Kunjaroenrut, Arttasid Udomvised, Tassawan Khayanchoomnoom, Watchara Meethaisong, Ketmookda Trairat

**HIVNAT, Thailand:** Thanyawee Puthanakit, Suvaporn Anugulruengkitt,  Wipaporn Natalie Songtaweesin, Torsak Bunupuradah, Naruporn Kasipong, Sararut Chanthaburanun, Apicha Mahanontharit, Kesdao Nanthapisal, Thidarat Jupimai, Thornthun Noppakaorattanamanee, Chutima Saisaengjan

**European Site Investigators:** Goethe University Frankfurt**, Germany:** Stephan Schultze-Strasser, Christoph Königs , **UKE Eppendorf, Germany:** Robin Kobbe, Ulf Schulze-Sturm, Felicia Mantkowski, Cornelius Rau, **Heartlands Hospital, UK:** Steve Welch, Jacqui Daglish, Laura Thrasyvoulou, Kate Gandhi, Yvonne Vaughan-Gordon, **Great Ormand Street Hospital, UK:** Delane Shingadia, Sophie Foxall, Judith Acero, Malgorzata Pasko-Szcech, Jacquie Flynn, **St Mary’s Hospital, UK:** Gareth Tudor-Williams, Amina Farhana Mehar, Caroline Foster, Sobia Mustafa, **Leicester Royal Infirmary, UK:** Srini Bandi, Jin Li, Jackie Philps, **Leeds General Infirmary, UK:** Sean O’Riordan, Dominique Barker, Richard Vowden, Maria Dowie **Kings College Hospital, UK:** Colin Ball Eniola Nsirim, Kathleen McClaughlin, **Hospital 12 de Octubre, Spain:** India Garcia, Pablo Rojo Conejo, Cristina Epalza, Luis Prieto Tato, Maite Fernandez **Hospital La Paz, Spain:** Luis Escosa Garcia, Maria José Mellado Peña, Talia Sainz Costa, **Hospital San Joan de Déu, Spain:** Claudia Fortuny Guasch, Antoni Noguera Julian, Carolina Estepa, Elena Bruno, Patricia Mendez Garcia, Alba Murciano Cabeza, Biobanco Gregorio Maranon, Maria Angeles Muñoz Fernandez, Jose Luis Jimenez, Coral Gomez Rico, **Centro Materno-infantile do Norte, Portugal:** Laura Marques, Carla Teixeira, Alexandre Fernandes, Rosita Nunes, Helena Nascimento, Andreia Padrao, Joana Tuna, Helena Ramos, Ana Constança Mendes, Helena Pinheiro, Ana Cristina Matos

**Local Site Monitors:** Flavia Kyomuhendo, Sarah Nakalanzi, Cynthia Mukisa Williams, Leora Sewnarain, Ntombenhle Ngcobo, Deborah Pako, Nompumelelo Yende, Jacky Crisp, Marlize Smuts, Benedictor Dube, Precious Chandiwana, Winnie Gozhora, Thidarat Jumpimai

*Substudies*

**PK substudies:** David Burger, Pauline Bollen, Angela Colbers, Hylke Waalewijn, Tom Jacobs

**Virology-immunology substudy:** Nigel Klein, Eleni Nastouli, Anita De Rossi, Maria Angeles Munoz Fernandez, Carlota Miranda, Moira Spyer,

**Social Science substudy and Youth Trial Board project:** Janet Seeley, Sarah Bernays, Stella Namukwaya, Zivai Mupambireyi, Magda Conway, Lungile Jafta, Mercy Shibemba

*Trial Committees*

**Independent Trial Steering Committee Members:** Ian Weller, Elaine Abrams, Tsitsi Apollo, Polly Clayden, Valériane Leroy

**Independent Data Monitoring Committee Members:** Anton Pozniak, Jane Crawley, Rodolphe Thiébaut, Helen McIlleron

**Endpoint Review Committee Members:** Alasdair Bamford, Hermione Lyall, Andrew Prendergast, Felicity Fitzgerald, Anna Goodman
